# Association between Brain-Derived Neurotrophic Factor and Lipid Profiles in Acute Ischemic Stroke Patients

**DOI:** 10.3390/ijms25042380

**Published:** 2024-02-17

**Authors:** Mayuri N. Tuwar, Wei-Hung Chen, Hsu-Ling Yeh, Chyi-Huey Bai

**Affiliations:** 1 School of Public Health, College of Public Health, Taipei Medical University, Taipei 106236, Taiwan; mayutuwar29@gmail.com; 2 Department of Neurology, Shin Kong Wu Ho-Su Memorial Hospital, Taipei 111045, Taiwan; 3 School of Medicine, College of Medicine, Taipei Medical University, Taipei 106236, Taiwan

**Keywords:** acute ischemic stroke, brain-derived neurotrophic factor (BDNF), cholesterol, LDL-C, HDL-C, TG

## Abstract

Ischemic stroke, the most prevalent form of stroke, leads to neurological impairment due to cerebral ischemia and affects 55–90% of the population. Brain-derived neurotrophic factor (BDNF) plays a crucial role in the central nervous system and regulates cardiometabolic risk factors, including lipids. This single-center study aimed to explore the relationship between lipid profiles and BDNF levels in 90 patients who had experienced AIS for the first time. The results show that the high BDNF group (≥3.227 ng/mL) had significantly higher HbA1C and TG levels; ratios of TC/HDL-C, LDL-C/HDL-C, and TG/HDL-C; and percentage of hyperlipidemia (60%) as well as lower levels of HDL-C, with an OR of 1.903 (95% CI: 1.187–3.051) for TG/HDL-C, 1.975 (95% CI: 1.188–3.284) for TC/HDL-C, and 2.032 (95% CI: 1.113–3.711) for LDL-C/HDL-C. Plasma BDNF levels were found to be significantly positively correlated with TG and negatively with HDL-C, with OR values of 1.017 (95% CI: 1.003–1.030) and 0.926 (95% CI: 0.876–0.978), respectively. TC/HDL-C, TG/HDL-C, and LDL-C/HDL-C ratios are associated with BDNF levels in AIS patients. The results also indicate that, in AIS patients, higher BDNF levels are associated with lower HDL and higher TG concentrations.

## 1. Introduction

Ischemic strokes are experienced by a significant portion of the population, estimated at between 55% and 90%, while hemorrhagic strokes affect 12% to 35% [1]. The primary cause of IS is cerebral ischemia, which leads to neurological impairment, and it the most prevalent subtype of stroke [1,2]. Dyslipidemia is a major risk factor for cardiovascular and cerebrovascular diseases and is characterized by abnormal levels of blood lipids, including elevated total cholesterol (TC), low-density lipoprotein cholesterol (LDL-C), and triglycerides (TG) and reduced high-density lipoprotein cholesterol (HDL-C) [3].

The National Cholesterol Education Program Adult Treatment Panel defines an HDL-C level under 40 mg/dL or TC level over 200 mg/dL as indicative of a high risk for ischemic heart disease [4]. Research in Northern Manhattan, New York, encompassing a diverse population, found a link between HDL-C levels and stroke risk, revealing that higher HDL-C levels, particularly in individuals aged 75 and above, were protective against IS across all racial groups [4,5,6]. Dyslipidemia is not only associated with atherosclerosis and stroke [7,8] but also impacts the smooth muscle and endothelial function of cerebral arteries [9]. Recent research suggests the development of vascular disorders is influenced by the pro-inflammatory properties of LDL-C and the anti-inflammatory traits of HDL-C [10].

While the association between the levels of individual lipids, particularly HDL-C, and AIS is well established, using lipid ratios provides a more comprehensive assessment of lipid metabolism and its relationship with BDNF levels. The use of lipid ratios to investigate the relationship between lipid profiles and AIS risk has been well established in the literature. The ratios of TC to HDL (TC/HDL), LDL to HDL (LDL/HDL), and TG to HDL (TG/HDL) are valuable indicators of atherogenic lipid profiles, which have been linked to increased cardiovascular risk, including stroke [11,12]. Lipid ratios provide insight into the overall lipid balance and are possibly a better representation of dyslipidemia-related risk factors than individual lipid parameters alone [13,14,15].

BDNF plays a vital role in the development, maintenance, and recovery of the central nervous system [16]. According to in vivo research, BDNF controls neurogenesis in the adult hippocampus and can thereby improve neuronal survival following injury or neurodegeneration in the developing brain [17,18,19]. BDNF is also present in peripheral tissues, such as those of adipose, muscle, and the cardiovascular system. While its exact role in these peripheral areas is still not fully understood, it appears to regulate glucose, lipids, and lipoproteins, which are all linked to cardiometabolic risk factors [20,21]. One relevant study closely evaluated the impact of free fatty acids (FFAs) and their inflammatory metabolites on the BDNF levels of 73 patients who had suffered an IS. The results showed a strong positive relationship between the levels of certain FFAs and BDNF [22]. However, the role of BDNF in regulating lipid profiles in IS patients is not well studied [23,24].

This study aimed to explore the relationship between the presence of BDNF and lipids, including TC, TG, LDL-C, and HDL-C, in patients who have experienced an AIS.

## 2. Results

### 2.1. Characteristics of Patients Based on BDNF Levels

Table 1 displays the characteristics of 90 AIS patients, categorized into two distinct groups according to their BDNF levels (45 patients in each group). One group consists of patients with BDNF levels ≤ 3.227 ng/mL, while the other includes those with BDNF levels ≥ 3.227 ng/mL. The table includes various health parameters, such as age, gender, and the prevalence of conditions like hyperlipidemia, diabetes, hypertension, and lifestyle factors (smoking and alcohol use). It also describes clinical measures such as systolic and diastolic blood pressure, white blood cell count, platelet count, glucose levels, and various cholesterol levels.

The average age was 67.19 years (±13.82) in the low BDNF group and 68.21 years (±12.00) in the high BDNF group, which is not statistically significant (*p* = 0.711). There were 68.9% and 66.7% males in the low and high BDNF groups, respectively, with a *p* value of 1.0, indicating no significant difference. Hyperlipidemia was found in 36.1% of the low BDNF group compared to 60.0% in the high BDNF group, corresponding to a statistically significant difference (*p* = 0.043). In contrast, diabetes was found in 63.6% of the low BDNF group and 71.9% of the high BDNF group, which was not significantly different (*p* = 0.598). Similarly, 75.6% and 68.2% of patients in the low and the high BDNF group, respectively, had hypertension, which was not significantly different (*p* = 0.486), whereas 40.0% and 29.7% in the low and the high BDNF group were smokers, also with no significant difference (*p* = 0.363). Around 23.3% and 20.0% of the low and the high BDNF group consumed alcohol, with no significant difference (*p* = 0.798), and the comparison of systolic (*p* = 0.585) and diastolic (*p* = 0.950) blood pressures, as well as white blood cell (WBC) counts (*p* = 0.767), between groups also showed there were no significant differences. However, there was a significant difference in platelet counts, with 262.98 (±111.68) in the low BDNF group and 210.68 (±76.98) in the high BDNF group (*p* = 0.013). Various blood parameters were compared, with significant differences found in TG, HDL-C, and TC/HDL-C, LDL-C/HDL-C, and TG/HDL-C ratios, but not in others. No significant differences in non-HDL-C and TyG index were found between the groups.

The key findings presented in the table include statistically significant differences in hyperlipidemia prevalence, platelet count, TG, high-density lipoprotein cholesterol, and the ratios of TC to HDL-C, LDL-C to HDL-C, and TG to HDL-C between the two groups.

Regarding the etiology of stroke, 28 patients (31.1%) were identified with large artery atherosclerosis, 3.3% with small artery atherosclerosis, 12 patients (13.3%) with cardio-embolism, 3.3% with determinable causes, and 11 patients (12.2%) with indeterminate causes.

### 2.2. Age and Sex-Adjusted Odds Ratios

The data in Table 2 show that the variables HDL-C, TG, TG/HDL-C, TC/HDL-C, LDL-C/HDL-C, and hyperlipidemia are significantly associated with higher levels of BDNF in AIS patients, while others such as TC, LDL-C, non-HDL-C, glucose, diabetes mellitus, and blood pressure are not significantly associated.

HDL-C shows a significant association, with an OR of 0.926 (95% CI: 0.876–0.978, *p* < 0.01). TG has an OR of 1.017 (95% CI: 1.003–1.030, *p* < 0.05), indicating a significant association. A significant correlation was seen between TG to HDL-C ratio, with an OR of 1.903 (95% CI: 1.187–3.051, *p* < 0.01). TC/HDL-C also demonstrates a significant association, with an OR of 1.975 (95% CI: 1.188–3.284, *p* < 0.01). LDL-C/HDL-C indicates a significant association, with an OR of 2.032 (95% CI: 1.113–3.711, *p* < 0.05). HbA1C shows a borderline significant association with an OR of 1.386 (95% CI: 0.983–1.953, *p* = 0.063).

### 2.3. Multivariate Logistic Regression

The results for the multivariate logistic regression of BDNF biomarkers and lipid profile, indicating risk of AIS, are shown in Table 3. In the first model, adjustments were made for age, sex, and HbA1C levels. These factors are included in the second model along with platelets. For the third and most extensive model, SBP, DBP, smoking status, alcohol consumption, and platelet count were added as adjustment factors. TC/HDL-C and TG/HDL-C ratios both showed significant associations with the risk of AIS across all models. Notably, the TC/HDL-C ratio demonstrated an odds ratio of 3.14 (95% CI: 1.31–7.54; *p* = 0.010) in the most extensive model, highlighting a substantially elevated risk. Similarly, the TG/HDL-C ratio demonstrated a significant correlation with stroke risk, with the OR peaking at 2.53 (95% CI: 1.24–5.17; *p* = 0.011). The LDL-C/HDL-C ratio exhibited borderline significance in the first two models but statistical significance in the third model, with an odds ratio of 3.02 (95% CI: 1.19–7.66; *p* = 0.020), indicating an increased risk of stroke. Hyperlipidemia did not show significant associations in any model, suggesting that the specific individual lipid ratios may be more predictive of stroke risk than the general condition of hyperlipidemia. 

### 2.4. Correlation between Serum BDNF Levels and Lipid Parameters

**Figure 1:**
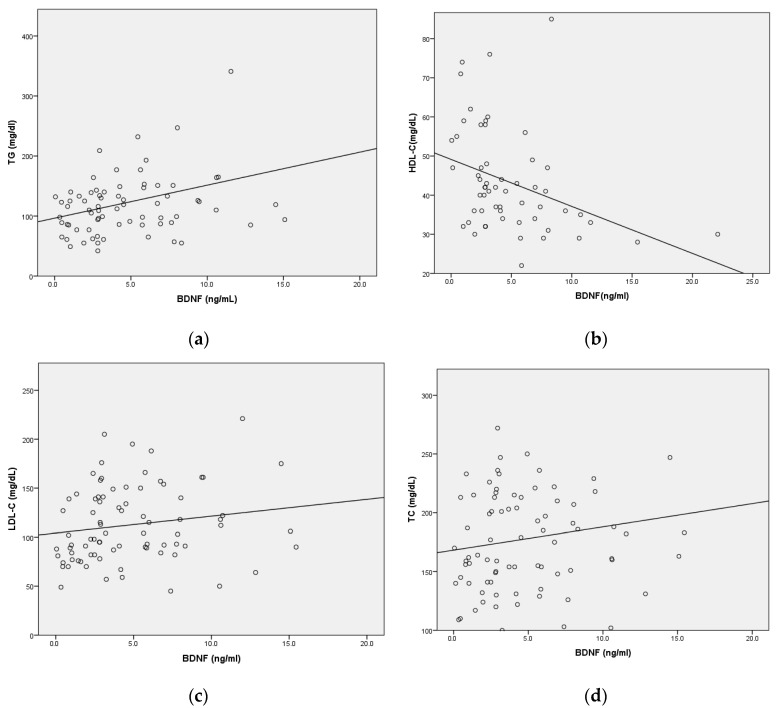
The correlation between serum BDNF levels and lipid parameters (TG, HDL, LDL, and TC): (**a**) TG (r = 0.301, B = 0.017, F = 7.19, *p* = 0.009), (**b**) HDL (r = −0.358, B = −0.107, F = 3.86, *p* = 0.006), (**c**) LDL (r = 0.184, B = 0.020, F = 2.78, *p* = 0.099), and (**d**) TC (r = 0.169, B = 0.014, F = 2.31, *p* = 0.133).

Figure 1 presents a scatter plot analyzing the correlations between plasma BDNF levels and various lipid parameters, including TG, HDL-C, LDL-C, and TC. The analysis reveals a significant positive correlation between plasma BDNF levels and TG, implying that elevated BDNF levels are linked to higher triglyceride levels. Conversely, a significant negative correlation is observed between plasma BDNF levels and HDL-C, indicating that higher levels of BDNF are associated with lower concentrations of HDL-C. However, the data for LDL-C and TC do not demonstrate any significant correlations with plasma BDNF levels.

## 3. Discussion

In our present study of stroke patients, we found that the high BDNF group (≥3.227 ng/mL) had significantly higher levels of HbA1C and TG; ratios of TC/HDL-C, LDL-C/HDL-C, and TG/HDL-C; and percentages of hyperlipidemia (60%), as well as lower levels of HDL-C and platelets. The ratios of TC/HDL-C, TG/HDL-C, and LDL-C/HDL-C showed significant associations with the risk of AIS when adjusted for multiple factors (age, sex, HbA1C, SBP, DBP, smoking, alcohol, and platelets). A positive correlation with TG levels and a negative correlation with HDL-C levels were observed, with LDL-C and TC showing weaker correlations. This indicates that lipid metabolism might play a crucial role in IS, as mediated through BDNF. Researchers have found a link between BDNF and lipid metabolism, confirming previous findings that BDNF levels are strongly positively correlated with TC, TG, and LDL, which suggest that BDNF is involved in the management of dyslipidemia [25,26,27]. One study showed that dyslipidemia is an important risk factor for stroke, and numerous studies have investigated the link between high cholesterol and stroke risk [28].

The significance of cholesterol originating from glial cells in the development of synapses has been established, and inhibiting cholesterol biosynthesis impacts the development of dendrites and axons in neurons within the cortical and hippocampal regions [29,30]. Findings from an electrophysiological experiment indicate that cholesterol synthesis, dependent on BDNF, contributes to the maturation of a readily releasable pool of synaptic vesicles. This suggests that BDNF, through its influence on cholesterol biosynthesis, is a crucial factor in the development of synapses [31]. BDNF boosts cholesterol biosynthesis by activating TrkB, leading to an elevation observed in rafts alongside an increase in presynaptic proteins. This implies that BDNF regulates the quantity of cholesterol specifically in presynaptic regions [31]. Signaling through TrkB in cholesterol-rich lipid rafts is crucial for the functioning of BDNF [32,33].

Another study investigated the relationship between BDNF, glucose, and lipid profile in Parkinson’s disease (PD) patients compared to healthy controls, finding that BDNF is a predictor of varying percentages of different lipid profile components but not glucose levels. Significant differences in certain lipid profile components were found between low BDNF and high BDNF groups, highlighting the importance of BDNF in lipid metabolism in the context of PD. However, as BDNF was not found to be a significant predictor of glucose levels, the findings suggest that alterations in BDNF might instead be linked to changes in the lipid metabolism of PD patients, offering new perspectives for understanding and managing the disease [23].

Elevated triglyceride levels have been significantly associated with an increased risk of IS. These high levels may indicate broader atherogenic and prothrombotic changes as well as abnormalities in the clotting–fibrinolytic system, which could further elevate stroke risk [34,35]. Elevated levels of blood glucose and cholesterol and low levels of HDL-C have been linked to a higher risk of atherosclerosis and stroke [36,37,38,39]. Amarenco et al. found that HDL-C level is inversely associated with stroke or carotid atherosclerosis, but more studies are needed to confirm this association [40]. A study based on participants from the UK Biobank found that a certain ratio of HDL-C to LDL-C was correlated with lower risks of myocardial infarction, all-cause mortality, hemorrhagic stroke, and IS. This suggests that HDL, often considered “good” cholesterol, might play a protective role in these conditions, including IS [41]. In a study conducted by You et al., the researchers examined the association between serum BDNF levels and lipid profiles among a Chinese population. Their findings revealed a negative correlation between serum BDNF levels and HDL-C levels [42]. This observation indicates that changes in lipid metabolism, specifically alterations in HDL-C levels, could influence BDNF levels, potentially affecting stroke prognosis. The researchers also found that higher serum BDNF levels were correlated with a decrease in poor prognosis following ischemic stroke. As a result, these findings suggest that BDNF can be used as a biomarker for assessing stroke prognosis and as a therapeutic target for improving stroke outcomes [43].

In our study, significant associations of BDNF (*p* < 0.05) are found with HDL-C, TG, and ratios of TG/HDL-C, TC/HDL-C, and LDL-C/HDL-C. This suggests that lipid profiles are notably associated with BDNF levels in stroke patients and implies that BDNF and lipid profiles might play a role in the pathophysiology of stroke and might be potential markers of its severity. An association was found between higher BDNF levels and increased TC. Elevated TC is another risk factor for cardiovascular issues, including stroke. High TG is linked to atherosclerosis and, consequently, an increased risk of stroke and heart attack. High LDL-C is a well-known contributor to plaque buildup in arteries, leading to increased stroke risk. However, determining the exact nature of this relationship and its clinical implications would require further investigation.

The relationship between serum lipid levels, such as of HDL-C, LDL-C, TG, and TC, and BDNF in IS patients has indeed been a topic of interest in medical research. While the individual roles of these serum lipids and BDNF in stroke pathology are well acknowledged, the precise nature of their interaction, especially in the context of IS, has not yet been fully elucidated. Research to date has primarily focused on the individual impacts of lipid profiles and BDNF in stroke. For example, elevated levels of certain lipids like LDL-C are known to be risk factors for IS, and BDNF has been recognized for its role in neuroprotection and neurodegeneration following stroke. However, the direct link between lipid levels and BDNF levels in stroke patients remains underexplored. Given the complexity of stroke pathology and the multifaceted roles of lipids and BDNF in the brain, further research in this area is necessary. Such research should ideally involve clinical studies that measure both lipid levels and BDNF in IS patients, aiming to uncover any correlations or causal relationships.

Our study has some limitations. First, the sample size is comparatively limited, and the study was undertaken in a single center, which limits the generalizability of the results. In addition, the blood samples were taken only once following the onset of IS symptoms.

## 4. Materials and Methods

For this study, we enrolled 90 patients with acute ischemic stroke (AIS) who were admitted to a stroke center in two separate time frames: from July 2014 to July 2015, and from November 2017 to September 2019. Inclusion as a case occurred only if the patient had no prior history of neurological or psychiatric conditions, including HD, stroke, transient ischemic attack (TIA), MS, PD, AD, or ALS. A patient was also eligible if it was their first AIS experience based on its clinical definition by the neurologist. Additionally, those who had infections or lacked complete baseline data were excluded based on specific criteria.

The structured questionnaire included data on admission demographics as well as the results of laboratory, radiographic, and clinical examination. One registered nurse with training was tasked with evaluating the patients’ functional results, while a neurologist certified for the study handled stroke diagnosis and neurological state. A stroke neurologist confirmed the stroke diagnosis based on the patient’s symptoms and brain imaging results from either magnetic resonance imaging (MRI) or computed tomography (CT) [44,45]. A trained, certified nurse assessed the functional outcome (used to collect information such as the smoking status, current medication for HTN or DM, and family history of diseases).

Diastolic blood pressure (DBP) equal to or exceeding 90 mmHg and systolic blood pressure (SBP) equal to or exceeding 140 mmHg are considered HTN [46,47,48]. For DM, fasting blood glucose levels must be 126 mg/dL or higher, along with HbA1c >6.5%. Hyperlipidemia involves TC levels greater than 200 mg/dL, LDL-C levels greater than 130 mg/dL, and TG greater than 150 mg/dL. In addition, all confirmed stroke patients underwent follow-up until their discharge or death. Furthermore, brain MRI, CT scans, or both were used to identify and evaluate the location and dimensions of the brain infarct lesion. Ischemic strokes were categorized into five subgroups using the Trial of Org 10,172 in Acute Stroke Treatment (TOAST) criteria, including large artery atherosclerosis, small vessel occlusion, cardioembolism, specific pathogenesis, and undetermined pathogenesis.

The study was accepted by Shin Kong Wu Ho-Su Memorial Hospital’s Investigational Review Board (IRB nos. 20140401R and 20170701R) and complies with the Declaration of Helsinki’s guidelines. Before beginning the study, each participant provided their written informed consent.

### 4.1. Blood Sampling

Blood samples from participants were taken at the time of admission to the emergency department if, at that time, the attending physician or neurologist suspected a stroke. The date and time of admission, blood draw, and medical procedures were documented in the enrollment paperwork. The sampling time duration was determined as the number of hours between the time of admission and the blood draw. Fasting blood samples were taken and centrifuged at 3000× *g* at room temperature for fifteen minutes within two hours of collection. Following this, they were divided into tubes containing plasma, serum, and buffy coats and stored at −80 °C until analysis.

### 4.2. Blood Lipids and Glucose

A laboratory test was used to measure fasting blood glucose levels, WBC, platelets, HbA1C, Hs-CRP, TC, LDL-C, HDL-C, and TG. Moreover, four alternative lipid profiles were examined in this study (non-HDL-C, TC/HDL-C, LDL-C/HDL-C, TG/HDL-C). As per the definition, non-HDL-C is equal to TC minus HDL-C (non-HDL-C = TC minus HDL-C). Additionally, the ratios of TC to HDL-C, LDL-C to HDL-C, and TG to HDL-C were determined as TC/HDL-C, LDL-C/HDL-C, and TG/HDL-C, respectively.

The formula for calculating the TyG index is TyG index = ln [fasting glucose (mg/dL) times fasting triglyceride (mg/dL)]/2 [17].

### 4.3. BDNF Measurement

An enzyme-linked immunosorbent test (ELISA) was used to quantify BDNF levels (Catalog No. DBD00, USA R&D Systems, Inc., Minneapolis, MN, USA). A Thermo ScientificTM MultiskanTM GO microplate spectrophotometer operating at 450~10 nm wavelength was used to quantify the color transformation. We measured the samples in duplicate to ensure accuracy. The lab technicians conducting the BDNF analysis were kept blind to the details of the study participants.

The minimal detectable dosage for this kit, <0.02 ng/mL, was determined to be zero. The threshold between high and low BDNF levels was determined by median (3.227 ng/mL).

### 4.4. Statistical Analysis

Analysis is based on the mean + standard deviation and the number of patients (percentage). Student’s *t*-tests and Mann–Whitney U tests were used for continuous variables, whereas Chi-square tests were used for categorical variables. Logistic regression analysis, with odds ratios (OR) and 95% confidence intervals (CI), was performed to evaluate the association between BDNF level and continuous lipid profiles, which includes TC, HDL-C, LDL-C, and TG in IS patients. Multivariable logistic regression was used to examine the association between lipid profile and BDNF. IBM SPSS software for Windows, version 23, was used. All *p*-values are two-sided, and the significance level was set at 0.05.

## 5. Conclusions

Our findings revealed a significant association between BDNF levels and specific lipid ratios such as TG/HDL-C, TC/HDL-C, and LDL-C/HDL-C. The findings also indicate that higher BDNF levels are associated with lower concentrations of HDL-C and higher concentrations of TG in AIS patients.

The study suggests that BDNF may play a role in lipid metabolism or serve as an indicator of lipid profile status in the context of IS. The significant associations with lipid ratios, in particular, highlight the potential of BDNF as a biomarker for stroke risk stratification and warrant further investigation into its clinical utility. Future research in this area could focus on elucidating the mechanistic pathways linking BDNF to lipid metabolism and exploring the potential of BDNF as a therapeutic target in stroke prevention and treatment.

## Figures and Tables

**Table 1:** Characteristics of 90 acute ischemic stroke patients categorized according to low and high BDNF levels.

	BDNF (*n* = 45) (≤3.227 ng/mL) N (%)/M ± SD	BDNF (*n* = 45) (≥3.227 ng/mL) N (%)/M ± SD	*p* Value
Age (y)	67.19 ± 13.82	68.21 ± 12.00	0.711
Gender (male)	31 (68.9)	30 (66.7)	1.0
Hyperlipidemia	13 (36.1)	24 (60.0)	0.043 *
Diabetes mellitus	21 (63.6)	23 (71.9)	0.598
Hypertension	34 (75.6)	30 (68.2)	0.486
Smoking	18 (40.0)	11 (29.7)	0.363
Alcohol	10 (23.3)	9 (20.0)	0.798
Systolic blood pressure (mmHg)	165.76 ± 45.79	160.84 ± 38.39	0.585
Diastolic blood pressure (mmHg)	90.93 ± 29.99	90.57 ± 24.18	0.950
White blood cells (10^3^/uL)	10.26 ± 4.11	10.54 ± 4.66	0.767
Platelet (10^3^/uL)	262.98 ± 111.68	210.68 ± 76.98	0.013 *
Glucose (mg/dL)	140.38 ± 56.82	151.65 ± 63.61	0.519
Hemoglobin A1c (%)	6.25 ± 1.48	7.06 ± 2.04	0.053
Triglycerides (mg/dL)	102.0 ± 36.79	144.08 ± 92.83	0.014 *
Total cholesterol (mg/dL)	174.34 ± 42.91	181.63 ± 54.07	0.508
High-density lipoprotein cholesterol (mg/dL)	48.14 ± 13.04	38.5 ± 11.63	0.005 **
Low-density lipoprotein cholesterol (mg/dL)	108.0 ± 35.59	117.0 ± 40.86	0.297
High-sensitivity C-reactive protein (mg/dL)	5.28 ± 5.64	6.16 ± 7.14	0.520
TC/HDL-C	3.87 ± 1.13	4.70 ± 1.18	0.008 **
LDL-C/HDL-C	2.45 ± 1.01	3.05 ± 0.98	0.028 *
TG/HDL-C	2.34 ± 1.15	3.88 ± 2.19	0.003 **
Non-HDL-C	134.76 ± 44.85	155.32 ± 61.80	0.114
TyG index	4.70 ± 0.32	4.83 ± 0.30	0.165

Note: *p* values refer to a two-sample *t*-test, Chi-square, or Fisher’s exact test. * *p* < 0.05 and ** *p* < 0.01.

**Table 2:** Age- and sex-adjusted odds ratios for acute ischemic stroke patients with high BDNF levels.

Variable	Adjusted OR (95% CI) ^a^	*p* Value
TC	1.003 (0.994–1.013)	0.496
LDL-C	1.006 (0.995–1.018)	0.280
HDL-C	0.926 (0.876–0.978)	0.006 **
TG	1.017 (1.003–1.030)	0.013 *
TG/HDL-C	1.903 (1.187–3.051)	0.008 **
TC/HDL-C	1.975 (1.188–3.284)	0.009 **
LDL-C/HDL-C	2.032 (1.113–3.711)	0.021 *
Non-HDL-C	1.008 (0.998–1.018)	0.120
TyG index	4.757 (0.584–38.778)	0.145
GLU	1.003 (0.993–1.013)	0.529
HbA1C	1.386 (0.983–1.953)	0.063
DM	1.439 (0.497–4.164)	0.502
Hyperlipidemia	2.665 (1.050–6.765)	0.039 *
SBP	0.997 (0.987–1.007)	0.561
DBP	1.000 (0.984–1.015)	0.961
HTN	0.670 (0.260–1.725)	0.407

Abbreviations: OR, odds ratio; CI, confidence interval; HDL-C, high-density lipoprotein cholesterol; LDL-C, low-density lipoprotein cholesterol; TC, total cholesterol; TG, triglycerides; HbA1C, hemoglobin A1c; GLU glucose; TyG index, triglyceride glucose index; DM, diabetes mellitus; SBP, systolic blood pressure; DBP, diastolic blood pressure; HTN, hypertension. ^a^ Adjusted for age and sex. * *p* < 0.05 and ** *p* < 0.01.

**Table 3:** Multivariate logistic regression of BDNF biomarkers and lipid profile indicating risk of acute ischemic stroke.

Variable	OR (95% CI) ^a^	*p* Value	OR (95% CI) ᵇ	*p* Value	OR (95% CI) ^c^	*p* Value
TC/HDL-C	1.76 (1.05–2.96)	0.034 *	1.78 (1.06–2.99)	0.031 *	3.14 (1.31–7.54)	0.010 *
TG/HDL-C	1.84 (1.14–2.97)	0.013 *	1.78 (1.11–2.86)	0.018 *	2.53 (1.24–5.17)	0.011 *
LDL-C/HDL-C	1.76 (0.95–3.28)	0.074	1.79 (0.96–3.35)	0.068	3.02 (1.19–7.66)	0.020 *
Hyperlipidemia	1.77 (0.65–4.83)	0.263	1.89 (0.67–5.33)	0.227	2.06 (0.65–6.53)	0.219

^a^—adjusted for age, sex, and HbA1C. ^b^—adjusted for age, sex, HbA1C, and platelets. ^c^—adjusted for age, sex, HbA1C, SBP, DBP, smoking, alcohol, and platelets. * *p* < 0.05.

## Data Availability

The data presented in this study are available from the corresponding author upon request. The data are not publicly available due to privacy restrictions.
